# Reflections as 2020 comes to an end: the editing and educational environment during the COVID-19 pandemic, the power of Scopus and Web of Science in scholarly publishing, journal statistics, and appreciation to reviewers and volunteers

**DOI:** 10.3352/jeehp.2020.17.44

**Published:** 2020-12-30

**Authors:** Sun Huh

**Affiliations:** Department of Parasitology and Institute of Medical Education, College of Medicine, Hallym University, Chuncheon, Korea; The Catholic University of Korea, Korea

## The COVID-19 pandemic and the educational environment

This year began with the spread of the coronavirus disease 2019 (COVID-19) pandemic, which has dramatically influenced all aspects of life for people throughout the world, including editors. A striking trend has been a marked increase in the number of submissions to journals. For instance, the number of submitted manuscripts increased by 42% in 2020, compared with an average expected increase of 11% during 2016–2019, in the neurosurgical, stroke neurology, and neurointerventional literature [1]. According to an analysis of a single journal, the *Journal of Animal Science and Technology*, “since May 2020, the article processing time has stably decreased. It is believed that the article processing time will continue to become shorter, as time spent at home will increase in the future” [2]. While there is a possibility of the frequent decline of review requests due to increased workload for online teaching if reviewers work in academic institutes. Publications on COVID-19 or SARS-CoV-2, the coronavirus that causes COVID-19, have expanded dramatically in various fields of science [3]. The *Journal of Educational Evaluation for Health Professions* (JEEHP) is not an exception. The number of submitted manuscripts has increased by 95% in 2020 (from 146 in 2019 to 285 as of December 25, 2020). JEEHP has also published some articles related to COVID-19, including an editorial [4], training materials [5], and a research article [6]. However, it is not fully evident how to interpret this increase in submissions. One possibility is that researchers may have more time to analyze their data because they now spend less time in meetings or travel. Another factor may be the dramatic increase in the frequency of online, or contactless meetings to offset the sharp decline in offline meetings. Online platforms make it easy for colleagues to meet with each other. Journal editing had already transitioned to online platforms, so there were no technical difficulties in handling the increased number of submitted manuscripts. According to a survey on the impact of COVID-19 on scholarly editors, some editors’ workload has increased, and the online-only publication of scholarly journals is predicted to accelerate after the COVID-19 pandemic [7].

There is still no substantive, evidence-based reason to think that the present situation will change until at least the end of 2021. Despite promising advances in vaccine development and chemotherapeutic drugs, large-scale trials will be necessary to determine their efficacy.

The COVID-19 pandemic has presented tremendous challenges for teachers and instructors at all levels of education. At my medical school, lectures have moved online in the form of video files or real-time lectures, but laboratory and clinical practice has continued to involve face-to-face activities from February to November of this year. Personally, as an instructor, I have found it difficult to implement problem-based learning or team-based learning (TBL) through an online platform. The issue is not that the transition to an online environment is likely to diminish students’ academic performance, since medical students in Korea are excellent at organizing their knowledge and acquiring skills; instead, the problem relates to the thought processes instilled by student-centered learning which enables the mutual learning and growth among peers. I wonder whether the transition to online learning may affect graduates’ competency, including social relationships. In Singapore, an online TBL platform was provided to teachers and students. In this system, the group of students could communicate through a separate communication platform, distinct from class-wide discussions [8]. The impact of these TBL classes on students’ academic performance remains to be ascertained as we continue to rely on online learning.

## The power of Scopus and Web of Science in scholarly publishing

I received a letter from an author who submitted a manuscript to the journal this August, stating that she stopped the procedure “because the journal is not indexed in Journal Citation Report (JCR).” This manuscript had already been revised twice according to the reviewers’ and editors’ comments—in other words, the manuscript was withdrawn at the stage of its final acceptance. Reviewers’ and editors’ time should not be wasted in this way. I have been frequently asked whether JEEHP is indexed in Science Citation Index Expanded (SCIE). The indexing and abstracting status of JEEHP is clearly presented on its website (https://www.jeehp.org/).

Authors generally read a journal’s aims and scope before submission and check whether the journal is indexed in international databases. Later, they check whether there is an article processing charge. JEEHP is a rare open-access journal in the category of “Education, Scientific Disciplines” indexed in PubMed Central, MEDLINE, Scopus, and Emerging Sources Citation Index without an article processing charge. No other journal in MEDLINE or SCIE focuses on high-stakes examinations (e.g., licensing examinations) in health education. I am not sure when the JEEHP will be listed in SCIE, as desired by many potential authors. If the 2-year impact factor reaches the 2nd quartile (i.e., over the 50th percentile) in the corresponding category, it will be re-evaluated by Clarivate Analytics. However, JEEHP still did not reach that status in 2020, as described below.

In a recent article, Tennant [8] sharply and incisively critiqued the role of commercial databases, including Scopus and Web of Science, in the global scientific enterprise. He suggested developing and using a public indexing database, which would include more scholarly journals globally because the research published in non-Western countries, in languages other than English, and in the fields of the arts, humanities, and social sciences is under-represented in large commercial databases. Tennant [8] argued that “the first simple step to resolve this problem is to simply stop all research from using either platform, and for users and institutes to stop subscribing to them”. However, it remains challenging to find supporters of public funding for this public project. One good model is PubMed Central, which is based on full-text JATS (Journal Article Tag Suite) XML [9]. By extending the language and research fields to include all other languages and research fields [10], the public indexing database project could be accomplished easily. Since PubMed Central is a full-text database, if publishers only added XML files of abstracts and references to the public indexing database, the effect would be to create new platform by some technical modifications.

## Journal metrics and statistics

**Fig. 1.**
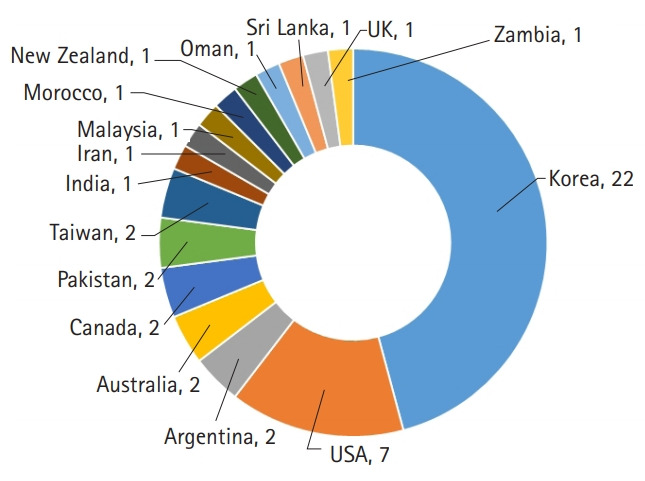
Countries of authors of *Journal of Educational Evaluation for Health Professions* in 2020.

**Fig. 2.**
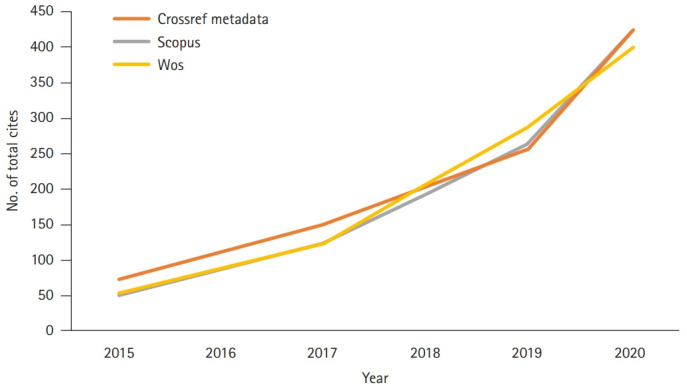
Total cites of *Journal of Educational Evaluation for Health Professions* in Crossref metadata, Scopus, and the Web of Science Core Collection (WOS) from 2015 to 2020.

**Fig. 3.**
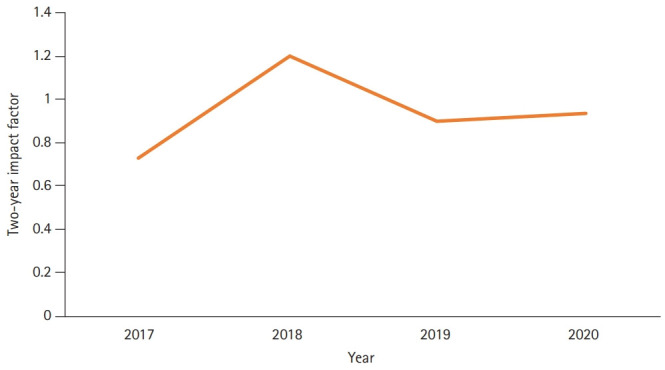
Two-year impact factor of *Journal of Educational Evaluation for Health Professions* calculated in the Web of Science Core Collection from 2017 to 2020.

The countries of the authors who published articles in JEEHP this year, as well as the total cites from Scopus, Web of Science Core Collection, and Crossref metadata, were analyzed. The 2-year impact factors calculated by the Web of Science were also calculated for recent years, although these are not official impact factors. In 2020, half of the articles were from Korean authors (Fig. 1). This large proportion derives from 3 editorials and 7 secondary or invited publications by Korean authors. The number of total cites from Crossref metadata, Scopus, and the Web of Science Core Collection increased dramatically from 254, 266, and 287 in 2019 to 423, 420, and 395 in 2020, respectively (Fig. 2). However, the calculated 2-year impact factor in the Web of Science Core Collection remained consistent, increasingly only very slightly from 0.90 in 2019 to 0.94 in 2020 (Fig. 3) [11]. This year’s 2-year impact factor corresponds to 26.2% (3rd quartile) in the JCR ranking of 2019. These trends show that older articles in JEEHP have been cited consistently and that this scientific field is not rapidly evolving.

This year, the journal published 8 secondary or invited publications. Five articles were from other journals: 1 from the *Korean Journal of the Academic Society of Nursing Education*, and 4 from the *Korean Medical Education Review*. One review was the report from the Korean Institute of Medical Education and Evaluation (KIMEE). The 2 educational/faculty development materials consisted of 1 guideline published in a booklet on extravasation and 1 training material containing the Korean Government’s guideline on COVID-19 (Table 1). The 5 reviews of the KIMEE’s history and works may furnish solid evidence of Korean medical society’s laborious work to promote the level of medical education to a top-tier level. I firmly believe that senior medical faculty members’ efforts are responsible for the excellent medical services provided in Korea during the COVID-19 pandemic.

Table 2 presents this year’s journal statistics. Although the number of submissions increased, the number of publications is not sharply different from that of the previous year. This reflects the low acceptance rate for unsolicited manuscripts (10.6%). Out of 275 unsolicited manuscripts, 230 (83.6%) were not sent for further review. The choice to send manuscripts to peer reviewers is made very carefully in order to maintain a sustainable workload for the editorial staff and reviewers, since it is very difficult to recruit reviewers. The acceptance rate of reviewed manuscripts was 62.8% (26/43).

Out of 44 publications this year, an editorial entitled “How to train health personnel to protect themselves from SARS-CoV-2 (novel coronavirus) infection when caring for a patient or suspected case” has been cited 50 times according to Crossref metadata [4]. I am happy that this editorial may contribute to health personnel’s safety. Furthermore, guidelines for distancing in daily life [5], and multi-professional simulation-based training on perceptions of safety and preparedness [6] will be helpful to overcome the present pandemic conditions.

## Appreciation to reviewers and volunteers

Due to the increased number of submissions, 74 reviewers from 19 countries were invited this year. Without their help, it would not be possible to publish the journal. JEEHP is deeply indebted to them. Below are the reviewers’ names and affiliations by country.

Australia: Michael Field, University of Sydney; Boaz Shulruf, University of New South Wales
Argentina: Raul Alfredo Borracci, Austral University
Canada: Oksana Babenko, University of Alberta; Armson Heather, University of Calgary
China: Yanhua Yi, Guangxi Medical University
Denmark: Steven Arild Wuyts Andersen, Rigshospitalet
India: Irfan Ali, Great Eastern Medical School and Hospital; Thalanjeri Padmini, Yenepoya Medical College; Khanna Vinay, Manipal Academy of Higher Education
Iran: Mohammad Esmaiel Hajinezhad, Iran University of Medical Science
Italy: Fabrizio Consorti, Università Sapienza of Rome
Korea: Duck Sun Ahn, World Federation for Medical Education; Hyung-Joon Ahn, Yonsei University; Ara Cho, Catholic University of Korea; Younyoung Choi, Hanyang Cyber University; Kyungsook Choi, Chung-Ang University; Cheol-Woon Chung, Catholic Kwandong University; Young Eun, Gyeongsang National University; Geum-Hee Jeong, Hallym University; Sunho Jung, Kyunghee University; Min Hyeok Kang, Busan Catholic University; Chul-Gyu Kim, Chungbuk National University; Sue Kim, Yonsei University; Sun Kim, Catholic University of Korea; Youngjon Kim, Wonkwang University; Ji-Woon Ko, Sunmoon University; Suk Bong Ko, Catholic University of Daegu; Young Hwan Lee, Yeungnam University; Kyunghee Lee, Shinhan University; Sujung Lee, Hallym University; Yonghee Lee, Korea University; Eunyoung Lim, Korean Institute Curriculum and Evaluation; Deuk-Sang Ma, Gangneung-Wonju National University; Mikyung Moon, Kyungpook National University; Younjae Oh, Hallym University; Bohyun Park, Changwon National University; Kwi Hwa Park, Gachon University; Dong Gi Seo, Hallym University; Yeonok Suh, Soonchunhyang University; Eun Young Suh; Seoul National University; Sanghee Yeo, Kyungpook National University; Mira Yun, Chung-Ang University
Mexico: Gonzalez Fernanda, Universidad Autónoma de Tamaulipas
Pakistan: Gardezi Syed Adeel Hussain, Combined Military Hospital; Khadija Qamar, Aga Khan University; Iqbal Waseem, College of Physicians and Surgeons Pakistan
Palestine: Ramzi Shawahna, An-Najah National University
Saudi Arabia: Mohammed Abdulrahman, King Saud bin Abdulaziz University for Health Science; Badr Alsayed, University of Tabuk; Amira Fraghely, Prince Sattam Bin Abdulaziz University
Spain: Jorge Riqueleme Galindo, University of Alicante
United Arab Emirates: Indira Kannan, Tawam Hospital
UK: Cleopatra Branch, Universities of Greenwich and Kent; Aaron Courtenay, Ulster University; Matt Homer, University of Leeds; Adam Rathbone, University of Newcastle
USA: Neeka Akhavan, University of Florida; Lynch Amanda, Oakland University; Erin Breitenbach, A.T. Still University; Stalvey Carolyn, University of Washington; Cathy Chang, Baylor College of Medicine; Eugene Jones, University of Texas Southwestern Medical Center; Myunghee Jun, University of Wisconsin-Green Bay; Linda Konecny, A.T. Still University; Sara Lolar, Wayne State University; Anna Miller-Fitzwater, Wake Forest Medical School; Takara Page, Ascension Health; Betty Del Rio Rodriguez, Baylor College of Medicine; Thongpriwan Vipavee, University of Wisconsin-Milwaukee; Hon Yuen, University of Alabama at Birmingham
Zambia: Aubrey Chichonyi Kalungia, University of Zambia; Ogah Nike, University of Zambia
Country unidentified: Manguiat Jose-Sebastian

Tom Huh, a graduate student of the Division of Life Sciences, College of Life Sciences and Biotechnology, Korea University, Seoul, Korea, voluntarily made the audio recordings of some abstracts.

## Artificial intelligence in editing

I will consider adopting artificial intelligence for editing and publishing [12]. Some programs have already appeared, and I have tried a few. However, it is still doubtful how much these programs will be helpful for editorial work. Nonetheless, I anticipate that with the development of artificial intelligence technology, many more convenient and useful programs will appear soon. A recent report addressed the ethical challenges regarding artificial intelligence in medicine from the perspective of scientific editing and peer review [13]. Particular consideration should be given to transparency of data and data sharing as the applications of artificial intelligence expand. Fortunately, JEEHP has adopted a data sharing policy. When big data are used for article preparation, transparency will be pursued during the review process.

## Salute from the editorial office to readers, authors, and reviewers

The editor’s job has been very hard for me since the beginning of the COVID-19 pandemic due to the rapid increase in submissions. As mentioned above, 230 manuscripts were rejected without peer review. Rejecting a submission is a serious decision, and the editor and the editorial team should read manuscripts thoroughly before making that choice. Furthermore, more reviewers should also devote themselves to reading manuscripts and writing reviews. Next year, we will do our best to select more scientifically described and well-written articles according to the journal’s style and format in order to save the reviewers time and to reduce their burden.

The COVID-19 pandemic may last until the end of 2021. We have trained medical and health personnel to combat this viral disease and to reduce its burden. Thanks to our collective efforts, the day will soon come when we will again be free from the present pandemic. I hope that all of the readers, authors, and reviewers of JEEHP will stay safe and be healthy in this pandemic period.

## Figures and Tables

**Table 1.** Secondary or invited articles in Journal of Educational Evaluation for Health Professions published in 2020

Publication type	Title	Article no.	Source
Review	Changes in the accreditation standards of medical schools by the Korean Institute of Medical Education and Evaluation from 2000 to 2019	2	Korean Institute of Medical Education and Evaluation report
Research article	Husserlian phenomenology in Korean nursing research: analysis, problems, and suggestions	13	*Journal of Korean Academic Society of Nursing Education*
Educational/faculty development materials	Rules and guidelines for distancing in daily life to control coronavirus disease 2019 in Korea: 3rd version, announced on July 3, 2020	20	The 3rd Korean-language version of rules and guidelines for distancing in daily life to coronavirus disease 2019 (COVID-19)
Educational/faculty development materials	Guidelines for the management of extravasation	21	Booklet
Review	History of the medical education accreditation system in Korea: implementation and activities in the early stages	29	*Korean Medical Education Review*
Review	Current trend of accreditation within medical education	30	*Korean Medical Education Review*
Review	Is accreditation in medical education in Korea an opportunity or a burden?	31	*Korean Medical Education Review*
Review	A proposal for the future of medical education accreditation in Korea	32	*Korean Medical Education Review*

**Table 2.** Journal statistics of manuscripts submitted to Journal of Educational Evaluation for Health Professions from January 1 to December 25, 2020

	Number	Content
Manuscripts submitted	286	
No. of commissioned manuscripts	11	Editorial, 3; educational/faculty development material, 2; review, 6
No. of unsolicited manuscripts	275	
Manuscripts under review or revision	2	Under the revision, 0; under review, 2
Manuscripts rejected without review	230	Unsuitable, 230; other reasons, 0
Manuscripts reviewed out of unsolicited manuscripts	43	Accepted and published, 27; rejected 13; withdrawal, 3
No. of publications out of 286 submitted manuscripts in 2020	38	Six articles published in 2020 were submitted in 2019
No. of publications out of 275 unsolicited manuscripts	27	
Acceptance rate overall (%)	13.3	38/284=0.133
Acceptance rate of unsolicited manuscripts (%)	10.6	29/273=0.106
Average time from submission to the first decision (day)	14	
Average time from submission to publication (day)	29	
Average time from acceptance to publication (day)	1	
